# Supplementary material for: Shifting headlines? Size trends of newsworthy fishes
Source: PeerJ. 2019 Feb 15;7:e6395. doi: 10.7717/peerj.6395 (PMC6378912; doi:10.7717/peerj.6395)
Supplement: Supplemental Information 1 — Lists of search terms and online sources used to find articles on ‘newsworthy’ fish. [file peerj-07-6395-s001.docx]

**Supplement 1 – Search terms and sources**

Shifting headlines? Trends in sizes of newsworthy fishes

Fiona T. Francis, Brett R. Howard, Trevor A. Branch, Adrienne E. Berchtold, Laís C.T. Chaves, Jillian C. Dunic, Brett Favaro, Kyla M. Jeffrey, Luis Malpica-Cruz, Natalie Maslowski, Jessica A. Schultz, Nicola S. Smith, and Isabelle M. Côté

**Table S1 Lists of search terms and online sources and used to find articles on ‘newsworthy’ fish.**

| **Search terms** | Fish **AND** monster; giant; caught; huge; mammoth; enormous; record; large; largest; big; gigantic; catch; rare; massive; whopper; world record; record breaking |
| --- | --- |
| **Databases** | **Web address** |
| Chronicling America (Library of Congress Online) | http://chroniclingamerica.loc.gov/ |
| LexisNexis Academic News | http://www.lexisnexis.com/hottopics/lNAcademic/ |
| Canadian Newsstand Database (Proquest) | http://www.proquest.com/ |
| Google News (search) | https://news.google.ca/ |
| Factiva | https://global.factiva.com |
| California Digital Newspaper Collection | http://cdnc.ucr.edu/cgi-bin/cdnc |
| Library of Congress | http://www.loc.gov/ |
| PapersPast | http://paperspast.natlib.govt.nz/cgi-bin/paperspast |
| New York Times Historical Database (Proquest) | http://www.proquest.com/ |
| Washington Post Database (Proquest) | http://www.proquest.com/ |
| **News aggregators** | |
| Zite | http://zite.com (defunct) |
| Google News | https://news.google.ca/ |
| Underwater Times | http://www.underwatertimes.com/ |
| Caribdaily | http://caribdaily.com/ |
| News360 (aggregator alert) | https://news360.com/ |
