# Supplementary material for: Shifting headlines? Size trends of newsworthy fishes
Source: PeerJ. 2019 Feb 15;7:e6395. doi: 10.7717/peerj.6395 (PMC6378912; doi:10.7717/peerj.6395)
Supplement: Supplemental Information 2 — Species group categories include: all epipelagic shark species targeted recreationally and/or commercially (Oceanic sharks); epipelagic teleost fishes typically targeted by recreational fishers (Pelagic gamefish); or species not regularly targeted by fishers but notable for the size and relative rarity (‘Charismatic megafish’). Species classified as NA were not analyzed by species group. Extinction risk categories (www.iucnredlist.org, 2016) include: all critically endangered, endangered, and vulnerable species (High); near threatened and least concern species (Low), and data-deficient and not-evaluated species together (Unknown). NTL = number of total length records, NWT = number of weight records. Species marked with an * indicate lengths were recorded as fork length, rather than total length. [file peerj-07-6395-s002.docx]

**Supplement 2 – Newsworthy species**

Shifting headlines? Trends in sizes of newsworthy fishes

Fiona T. Francis, Brett R. Howard, Trevor A. Branch, Adrienne E. Berchtold, Laís C.T. Chaves, Jillian C. Dunic, Brett Favaro, Kyla M. Jeffrey, Luis Malpica-Cruz, Natalie Maslowski, Jessica A. Schultz, Nicola S. Smith, and Isabelle M. Côté

**Table S2 List of the 75 fish species reported in news items, including species group and extinction risk subgroup categories**. Species group categories include: all epipelagic shark species targeted recreationally and/or commercially (Oceanic sharks); epipelagic teleost fishes typically targeted by recreational fishers (Pelagic gamefish); or species not regularly targeted by fishers but notable for the size and relative rarity (‘Charismatic megafish’). Species classified as NA were not analyzed by species group. Extinction risk categories (www.iucnredlist.org, 2016) include: all critically endangered, endangered, and vulnerable species (High); near threatened and least concern species (Low), and data-deficient and not-evaluated species together (Unknown). N_TL_ = number of total length records, N_WT_ = number of weight records. Species marked with an * indicate lengths were recorded as fork length, rather than total length.

| **Genus** | **Common name** | **Species group** | **Extinction risk** | **N_TL_** | **N_WT_** |
| --- | --- | --- | --- | --- | --- |
| *Acanthocybium solandri* | wahoo | Pelagic gamefish | Low | 1 | 1 |
| *Aetobatus narinari* | spotted eagle ray | Charismatic megafish | Low | 0 | 1 |
| *Albula vulpes* | bonefish | NA | Low | 0 | 1 |
| *Alopias superciliosus* | bigeye thresher | Oceanic shark | High | 0 | 1 |
| *Alopias vulpinus* | thresher | Oceanic shark | High | 5 | 5 |
| *Anarrhichthys ocellatus* | wolf eel | NA | Unknown | 1 | 0 |
| *Archosargus probatocephalus* | sheepshead | NA | Low | 1 | 1 |
| *Argyrosomus japonicus* | Japanese meagre | NA | Unknown | 0 | 1 |
| *Atractoscion nobilis* | white weakfish | NA | Low | 0 | 1 |
| *Bahaba taipingensis* | Chinese bahaba | NA | High | 1 | 1 |
| *Calamus bajonado* | jolthead porgy | NA | Low | 0 | 1 |
| *Carcharhinus brachyurus* | copper shark | Oceanic shark | Low | 1 | 1 |
| *Carcharhinus brevipinna* | spinner shark | Oceanic shark | Low | 1 | 1 |
| *Carcharhinus leucas* | bull shark | Oceanic shark | Low | 1 | 0 |
| *Carcharhinus obscurus* | dusky shark | Oceanic shark | High | 1 | 0 |
| *Carcharodon carcharias* | great white shark | Oceanic shark | High | 7 | 9 |
| *Cetorhinus maximus* | basking shark | Charismatic megafish | High | 3 | 2 |
| *Conger conger* | European conger | NA | Low | 0 | 1 |
| *Coryphaena hippurus* | common dolphinfish | Pelagic gamefish | Low | 0 | 1 |
| *Cynoscion nebulosus* | spotted weakfish | NA | Low | 1 | 1 |
| *Dasyatis centroura* | roughtail stingray | NA | Low | 1 | 1 |
| *Dipturus batis* | blue skate | NA | High | 5 | 5 |
| *Epinephelus daemelii* | saddletail grouper | NA | Low | 1 | 1 |
| *Epinephelus itajara* | Atlantic goliath grouper | NA | High | 1 | 2 |
| *Epinephelus lanceolatus* | giant grouper | NA | High | 1 | 3 |
| *Epinephelus nigritus* | Warsaw grouper | NA | High | 2 | 2 |
| *Epinephelus quinquefasciatus* | Pacific goliath grouper | NA | Unknown | 0 | 1 |
| *Gadus morhua* | Atlantic cod | NA | High | 3 | 8 |
| *Galeocerdo cuvier* | tiger shark | Oceanic shark | Low | 8 | 6 |
| *Heptranchias perlo* | seven gill shark | NA | Low | 0 | 1 |
| *Hippoglossus hippoglossus* | Atlantic halibut | NA | High | 4 | 8 |
| *Hippoglossus stenolepis* | Pacific halibut | NA | Unknown | 11 | 14 |
| *Istiompax indica** | black marlin | Pelagic gamefish | Unknown | 4 | 7 |
| *Isurus oxyrinchus* | shortfin mako | Oceanic shark | High | 14 | 16 |
| *Lamna nasus* | porbeagle | Oceanic shark | High | 1 | 1 |
| *Lates calcarifer* | barramundi | NA | Unknown | 1 | 0 |
| *Lophius americanus* | American angler | NA | Unknown | 1 | 0 |
| *Lutjanus cyanopterus* | Cubera snapper | NA | High | 1 | 1 |
| *Lutjanus sebae** | Emperor red snapper | NA | Unknown | 1 | 1 |
| *Makaira mazara* | Indo-Pacific blue marlin | Pelagic gamefish | Unknown | 2 | 5 |
| *Makaira nigricans* | blue marlin | Pelagic gamefish | High | 4 | 12 |
| *Mobula (Manta) birostris* | giant manta | Charismatic megafish | High | 2 | 7 |
| *Megachasma pelagios* | megamouth shark | Charismatic megafish | Low | 1 | 1 |
| *Megalops atlanticus* | tarpon | NA | High | 0 | 1 |
| *Mitsukurina owstoni* | goblin shark | NA | Low | 2 | 1 |
| *Mola mola* | ocean sunfish | Charismatic megafish | High | 8 | 10 |
| *Molva molva* | ling | NA | Unknown | 1 | 1 |
| *Morone saxatilis* | striped bass | NA | Low | 3 | 7 |
| *Nematistius pectoralis** | roosterfish | NA | Unknown | 1 | 0 |
| *Paralichthys californicus* | California flounder | NA | Low | 0 | 1 |
| *Paralichthys dentatus* | summer flounder | NA | Low | 1 | 1 |
| *Pogonias cromis* | black drum | NA | Low | 0 | 1 |
| *Pollachius virens* | saithe | NA | Unknown | 0 | 1 |
| *Pomatomus saltatrix* | bluefish | NA | High | 1 | 1 |
| *Prionace glauca* | blue shark | Oceanic shark | Low | 3 | 3 |
| *Pristis pectinata* | smalltooth sawfish | NA | High | 1 | 1 |
| *Pristis perotteti* | largetooth sawfish | NA | High | 0 | 1 |
| *Pristis pristis* | common sawfish | NA | High | 1 | 1 |
| *Pterois volitans* | red lionfish | NA | Unknown | 1 | 1 |
| *Regalecus glesne* | giant oarfish | NA | Low | 3 | 0 |
| *Rhincodon typus* | whale shark | Charismatic megafish | High | 9 | 6 |
| *Rhinobatos productus* | shovelnose guitarfish | NA | Low | 1 | 0 |
| *Sciaenops ocellatus* | red drum | NA | Low | 0 | 1 |
| *Scomberomorus commerson** | Narrow barred Spanish mackerel | Pelagic gamefish | Low | 2 | 1 |
| *Scophthalmus maximus* | turbot | NA | Unknown | 0 | 1 |
| *Sebastes borealis* | shortraker rockfish | NA | Unknown | 1 | 1 |
| *Seriola lalandi* | yellowtail amberjack | NA | Low | 1 | 3 |
| *Somniosus microcephalus* | Greenland shark | NA | Low | 2 | 2 |
| *Sphyrna mokarran* | great hammerhead | Oceanic shark | High | 3 | 3 |
| *Stereolepis gigas* | giant seabass | NA | High | 2 | 8 |
| *Tautoga onitis* | tautog | NA | High | 0 | 1 |
| *Thunnus albacares* | yellowfin tuna | Pelagic gamefish | Low | 0 | 5 |
| *Thunnus orientalis** | Pacific bluefin tuna | Pelagic gamefish | High | 2 | 22 |
| *Thunnus thynnus* | Atlantic bluefin tuna | Pelagic gamefish | High | 4 | 25 |
| *Xiphias gladius** | swordfish | Pelagic gamefish | Low | 6 | 5 |
