# Supplementary material for: Shifting headlines? Size trends of newsworthy fishes
Source: PeerJ. 2019 Feb 15;7:e6395. doi: 10.7717/peerj.6395 (PMC6378912; doi:10.7717/peerj.6395)
Supplement: Supplemental Information 3 — Results of quantile regression analyses for newsworthy fishes in our database. Quantile regressions highlighted in grey did not meet the minimum sample size ratio required (ratio > 5) and were therefore not shown in respective figures. Quantile regressions highlighted in bold met the minimum sample size threshold and were significant. [file peerj-07-6395-s003.docx]

**Supplement 4 – Quantile regression results**

**Shifting headlines? Trends in sizes of newsworthy fishes**

Fiona T. Francis, Brett R. Howard, Trevor A. Branch, Adrienne E. Berchtold, Laís C.T. Chaves, Jillian C. Dunic, Brett Favaro, Kyla M. Jeffrey, Luis Malpica-Cruz, Natalie Maslowski, Jessica A. Schultz, Nicola S. Smith, and Isabelle M. Côté

**Table S3**. Results of quantile regressions of (a) relative length and (b) weight vs year, for all newsworthy fishes in our database. Quantile regressions highlighted in grey did not meet the minimum sample size ratio required (ratio > 5) and were therefore not shown in Fig. 1. Quantile regressions highlighted in bold met the minimum sample size threshold and were significant.

Quantile Slope CI slope p Ratio

(tau)

(a) Relative length

0.10 -0.0006 -0.0023 – 0.0010 0.45 15.0

0.15 0.0000 -0.0016 – 0.0016 1.00 22.5

0.20 -0.0007 -0.0021 – 0.0008 0.36 30.0

0.25 -0.0008 -0.0020 – 0.0003 0.15 37.5

0.50 -0.0002 -0.0009 – 0.0004 0.53 75.0

0.75 -0.0006 -0.0016 – 0.0005 0.30 37.5

**0.80 -0.0013 -0.0022 – -0.0003 0.01 30.0**

**0.85 -0.0010 -0.0019 – -0.0002 0.02 22.5**

0.90 -0.0006 -0.0012 – 0.0000 0.06 15.0

(b) Relative weight

0.10 0.0006 -0.0006 – 0.0017 0.32 23.7

0.15 0.0008 -0.0007 – 0.0023 0.31 35.6

0.20 0.0005 -0.0008 – 0.0018 0.45 47.4

0.25 0.0000 -0.0013 – 0.0012 0.92 59.3

0.50 0.0004 -0.0011 – 0.0019 0.58 118.5

0.75 0.0003 -0.0008 – 0.0015 0.54 59.3

0.80 0.0007 -0.0007 – 0.0022 0.30 47.4

0.85 0.0011 -0.0009 – 0.0031 0.26 35.6

0.90 -0.0001 -0.0024 – 0.0021 0.91 23.7

**Table S4**. Results of quantile regressions of relative **length** vs year for groups of fishes: (a) Pelagic gamefish, (b) oceanic sharks, and (c) charismatic megafishes. Quantile regressions highlighted in grey did not meet the minimum sample size required (ratio > 5) and were therefore not shown in Fig. 2. Quantile regressions highlighted in bold met the minimum sample size threshold and were significant.

Quantile Slope CI slope p Ratio

(tau)

(a) Pelagic gamefish

0.10 -0.0015 -0.0062 – 0.0033 0.52 2.5

0.15 -0.0012 -0.0046 – 0.0023 0.49 3.8

0.20 -0.0016 -0.0041 – 0.0009 0.20 5.0

0.25 -0.0015 -0.0034 – 0.0005 0.14 6.2

0.50 -0.0007 -0.0027 – 0.0013 0.50 12.5

0.75 -0.0013 -0.0037 – 0.0011 0.28 6.2

0.80 -0.0010 -0.0036 – 0.0015 0.41 5.0

0.85 -0.0007 -0.0035 – 0.0020 0.58 3.8

0.90 -0.0004 -0.0030 – 0.0022 0.76 2.5

(b) Oceanic sharks

0.10 0.0002 -0.0017 – 0.0021 0.81 4.5

0.15 0.0001 -0.0016 – 0.0019 0.86 6.8

0.20 0.0006 -0.0015 – 0.0027 0.57 9.0

0.25 0.0008 -0.0017 – 0.0033 0.53 11.2

0.50 0.0012 -0.0016 – 0.0040 0.41 22.5

0.75 -0.0002 -0.0033 – 0.0029 0.90 11.2

0.80 -0.0002 -0.0025 – 0.0022 0.88 9.0

0.85 -0.0014 -0.0045 – 0.0017 0.38 6.8

0.90 -0.0011 -0.0050 – 0.0027 0.56 4.5

(c) Charismatic megafish

0.10 -0.0037 -0.0061 – -0.0012 0.005 2.3

0.15 -0.0035 -0.0006 – -0.0011 0.007 3.4

0.20 -0.0035 -0.0056 – -0.0014 0.002 4.6

**0.25 -0.0034 -0.0053 – -0.0014 0.002 5.8**

0.50 -0.0029 -0.0064 – 0.0005 0.09 11.5

0.75 -0.0020 -0.0049 – 0.0009 0.16 5.8

0.80 -0.0017 -0.0048 – 0.0014 0.28 4.6

0.85 -0.0035 -0.0066 – -0.0005 0.03 3.5

0.90 -0.0043 -0.0072 – -0.0014 0.005 2.3

**Table S5**. Results of quantile regressions of relative **weight** vs year for groups of fishes: (a) Pelagic gamefish, (b) oceanic sharks, and (c) charismatic megafishes. Quantile regressions highlighted in grey did not meet the minimum sample size ratio required (ratio > 5) and were therefore not shown in Fig. S2. Quantile regressions highlighted in bold met the minimum sample size threshold and were significant.

Quantile Slope CI slope p Ratio

(tau)

(a) Pelagic gamefish

**0.10 0.0022 0.0007 – 0.0037 0.04 27.3**

**0.15 0.0017 0.0004 – 0.0030 0.01 10.9**

**0.20 0.0019 0.0004 – 0.0035 0.01 14.6**

**0.25 0.0020 0.0000 – 0.0040 0.05 18.2**

0.50 0.0019 -0.0002 – 0.0041 0.08 36.5

0.75 0.0021 -0.0002 – 0.0044 0.07 18.2

0.80 0.0017 -0.0016 – 0.0050 0.31 14.6

0.85 0.0034 -0.0005 – 0.0004 0.09 11.0

0.90 0.0031 -0.0014 – 0.0076 0.18 7.3

(b) Oceanic sharks

0.10 -0.0022 -0.0092 – 0.0049 0.54 4.6

0.15 -0.0014 -0.0008 – 0.0053 0.68 6.9

0.20 -0.0009 -0.0062 – 0.0044 0.75 9.2

0.25 -0.0005 -0.0061 – 0.0051 0.87 11.5

0.50 0.0001 -0.0026 – 0.0027 0.97 23.0

0.75 0.0009 -0.0035 – 0.0054 0.67 11.5

0.80 0.0010 -0.0030 – 0.0050 0.61 9.2

0.85 0.0027 -0.0011 – 0.0064 0.16 6.9

0.90 0.0028 -0.0021 – 0.0076 0.26 4.6

(c) Charismatic megafish

0.10 -0.0013 -0.0031 – 0.0006 0.16 2.7

0.15 -0.0020 -0.0041 – 0.0002 0.07 4.0

**0.20 -0.0021 -0.0042 – -0.0001 0.04 5.4**

0.25 -0.0017 -0.0039 – 0.0004 0.11 6.8

0.50 -0.0029 -0.0064 – 0.0007 0.11 13.5

0.75 -0.0018 -0.0064 – 0.0028 0.42 6.8

0.80 -0.0025 -0.0075 – 0.0026 0.33 5.4

0.85 -0.0013 -0.0072 – 0.0046 0.65 4.1

0.90 0.0022 -0.0033 – 0.0077 0.41 2.7

**Table S6**. Results of quantile regressions of relative **length** vs year for subgroups of fishes at (a) unknown risk, (b) low risk, and (c) high risk, according to their IUCN threat status (see Methods). Quantile regressions highlighted in grey did not meet the minimum sample size ratio required (ratio > 5) and were therefore not shown in Fig. S3. Quantile regressions highlighted in bold met the minimum sample size threshold and were significant.

Quantile Slope CI slope p Ratio

(tau)

(a) Unknown risk

0.10 0.0062 -0.0035 – 0.0158 0.20 2.0

0.15 0.0063 -0.0025 – 0.0151 0.15 3.0

0.20 0.0065 -0.0019 – 0.0149 0.12 4.0

0.25 0.0032 -0.0054 – 0.0119 0.44 5.0

0.50 -0.0006 -0.0067 – 0.0056 0.85 10.0

0.75 0.0001 -0.0036 – 0.0038 0.97 5.0

0.80 0.0002 -0.0038 – 0.0042 0.92 4.0

0.85 0.0004 -0.0036 – 0.0044 0.83 3.0

0.90 0.0007 -0.0026 – 0.0040 0.67 2.0

(b) Low risk

0.10 -0.0007 -0.0024 – 0.0010 0.42 4.1

0.15 -0.0014 -0.0033 – 0.0006 0.16 6.1

0.20 -0.0010 -0.0033 – 0.0012 0.36 8.2

0.25 -0.0013 -0.0034 – 0.0007 0.20 10.2

0.50 -0.0013 -0.0027 – 0.0001 0.06 20.5

0.75 -0.0002 -0.0019 – 0.0015 0.83 10.2

0.80 -0.0001 -0.0022 – 0.0020 0.94 8.2

0.85 -0.0005 -0.0027 – 0.0017 0.63 6.2

0.90 -0.0006 -0.0027 – 0.0014 0.53 4.1

(c) High risk

0.10 -0.0007 -0.0036 – 0.0022 0.65 8.5

0.15 0.0001 -0.0022 – 0.0024 0.94 12.8

0.20 -0.0010 -0.0027 – 0.0007 0.27 17.0

0.25 -0.0008 -0.0021 – 0.0004 0.19 21.2

0.50 0.0001 -0.0015 – 0.0017 0.89 42.5

**0.75 -0.0014 -0.0028 – 0.0000 0.05 21.2**

**0.80 -0.0013 -0.0026 – -0.0001 0.03 17.0**

**0.85 -0.0016 -0.0024 – -0.0007 0.001 12.8**

**0.90 -0.0017 -0.0028 – -0.0005 0.005 8.5**

**Table S7**. Results of quantile regressions of relative **weight** vs year for subgroups of fishes at (a) unknown risk, (b) low risk, and (c) high risk, according to their IUCN threat status (see Methods). Quantile regressions highlighted in grey did not meet the minimum sample size required (ratio > 5) and were therefore not shown in Fig. S3. Quantile regressions highlighted in bold met the minimum sample size threshold and were significant.

Quantile Slope CI slope p Ratio

(tau)

(a) Unknown risk

0.10 0.0038 -0.0057 – 0.0132 0.22 2.6

0.15 0.0042 -0.0039 – 0.0123 0.30 3.9

0.20 0.0043 -0.0037 – 0.0122 0.28 5.2

0.25 0.0048 -0.0018 – 0.0114 0.15 6.5

0.50 0.0031 -0.0073 – 0.0136 0.54 13.0

0.75 0.0046 -0.0110 – 0.0203 0.55 6.5

0.80 0.0053 -0.0094 – 0.0201 0.47 5.2

0.85 0.0007 -0.0162 – 0.0176 0.93 3.9

0.90 -0.0010 -0.0175 – 0.0155 0.90 2.6

(b) Low risk

0.10 0.0007 -0.0008 – 0.0023 0.35 5.2

0.15 0.0008 -0.0009 – 0.0024 0.36 7.8

0.20 0.0008 -0.0011 – 0.0028 0.38 10.4

0.25 0.0015 -0.0009 – 0.0040 0.21 13.0

0.50 0.0007 -0.0020 – 0.0034 0.61 26.0

0.75 0.0002 -0.0031 – 0.0035 0.92 13.0

0.80 0.0009 -0.0028 – 0.0047 0.63 10.4

0.85 0.0014 -0.0029 – 0.0056 0.52 7.8

0.90 0.0000 -0.0039 – 0.0039 1.00 5.2

(c) High risk

0.10 0.0002 -0.0014 – 0.0018 0.79 15.1

0.15 -0.0007 -0.0030 – 0.0015 0.52 22.6

0.20 -0.0001 -0.0021 – 0.0019 0.92 30.6

0.25 -0.0004 -0.0019 – 0.0010 0.57 37.8

0.50 -0.0002 -0.0020 – 0.0017 0.86 75.5

0.75 0.0004 -0.0008 – 0.0017 0.53 37.8

0.80 0.0007 -0.0007 – 0.0021 0.33 30.2

0.85 0.0014 -0.0001 – 0.0030 0.07 22.7

0.90 0.0016 -0.0004 – 0.0036 0.11 15.1
