# Supplementary material for: Shifting headlines? Size trends of newsworthy fishes
Source: PeerJ. 2019 Feb 15;7:e6395. doi: 10.7717/peerj.6395 (PMC6378912; doi:10.7717/peerj.6395)
Supplement: Supplemental Information 4 — Length of charismatic megafishes in printed news headlines from 1869 to 2015, relative to the maximum species-specific weight, (A) when all Mola are assumed to be Mola mola, and (B) when all Mola that are smaller than the minimum size of Mola tecta (242 cm) are assumed to be Mola tecta. Lines represent quantiles regressions that met a minimum sample size (see Methods). Significant quantile regressions are shown as solid lines; non-significant (p < 0.05) quantiles regressions are shown in dotted lines. The dashed line is the 50th quantile. [file peerj-07-6395-s004.docx]

**Supplement 3 – Sensitivity analysis of taxonomic uncertainty**

**Shifting headlines? Trends in sizes of newsworthy fishes**

Fiona T. Francis, Brett R. Howard, Trevor A. Branch, Adrienne E. Berchtold, Laís C.T. Chaves, Jillian C. Dunic, Brett Favaro, Kyla M. Jeffrey, Luis Malpica-Cruz, Natalie Maslowski, Jessica A. Schultz, Nicola S. Smith, and Isabelle M. Côté


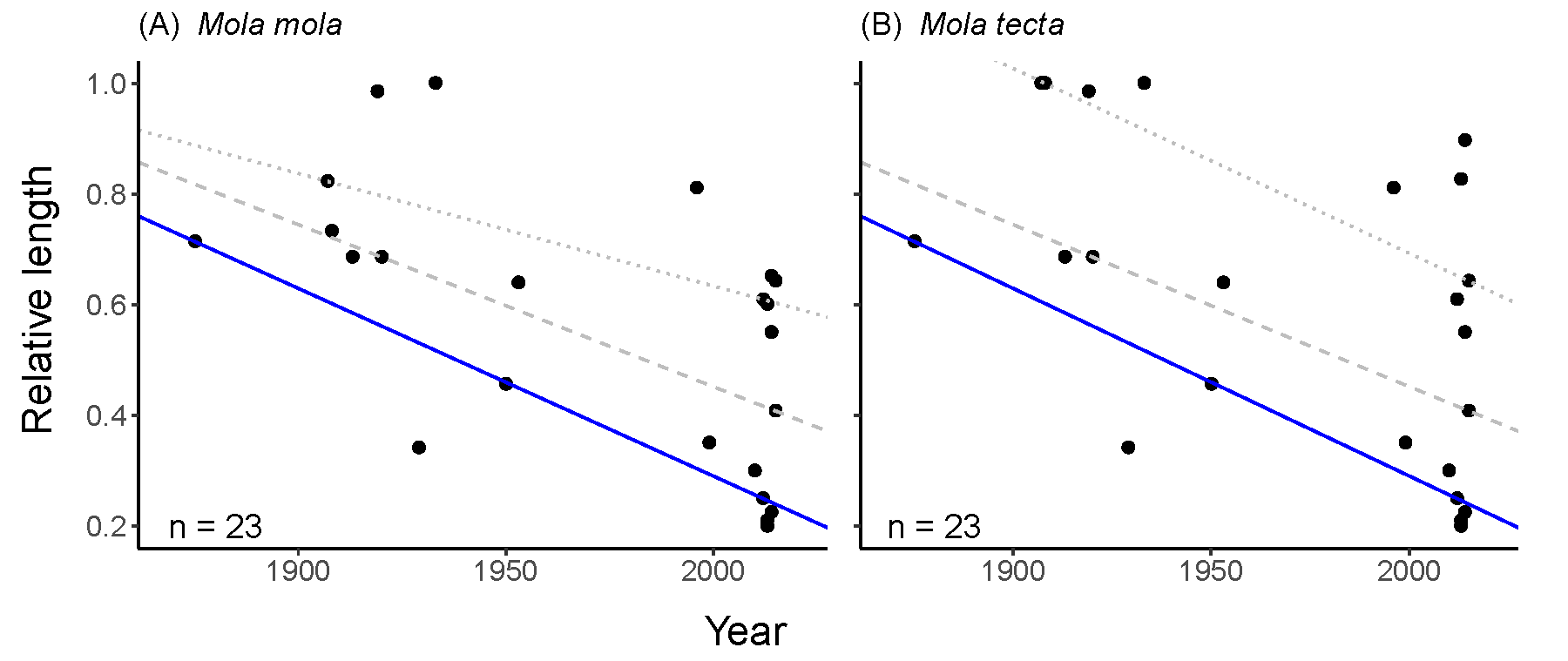


**Figure S1. Sensitivity to taxonomic uncertainty of trends in relative length of charismatic megafishes reported as being exceptional large in printed news headlines over time.** Length of charismatic megafishes in printed news headlines from 1869 to 2015, relative to the maximum species-specific weight, (A) when all *Mola* are assumed to be *Mola mola*, and (B) when all *Mola* that are smaller than the minimum size of *Mola tecta* (242 cm) are assumed to be *Mola tecta*. Lines represent quantiles regressions that met a minimum sample size (see Methods). Significant quantile regressions are shown as solid lines; non-significant (p < 0.05) quantiles regressions are shown in dotted lines. The dashed line is the 50^th^ quantile.
