# Supplementary figures and images for: Shifting headlines? Size trends of newsworthy fishes
Source: PeerJ. 2019 Feb 15;7:e6395. doi: 10.7717/peerj.6395 (PMC6378912; doi:10.7717/peerj.6395)

(A) Pelagic gamefish

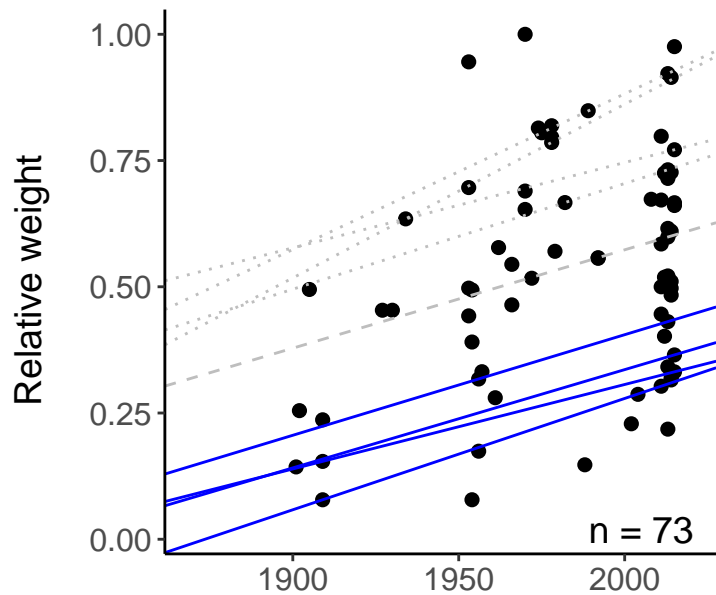

(B) Oceanic sharks

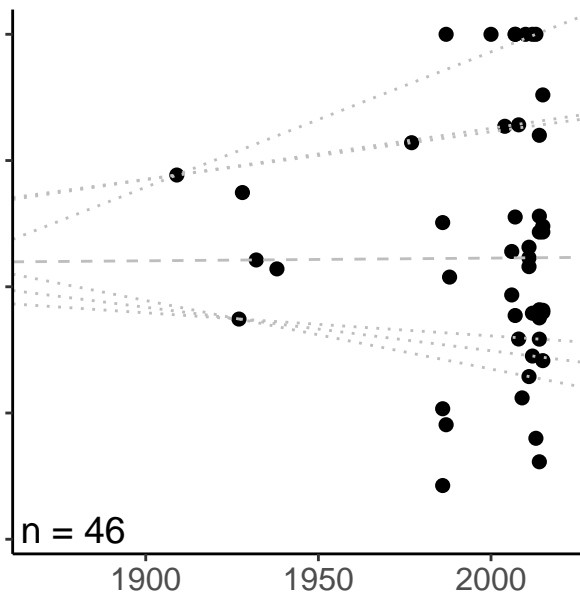

(C) Charismatic megafish

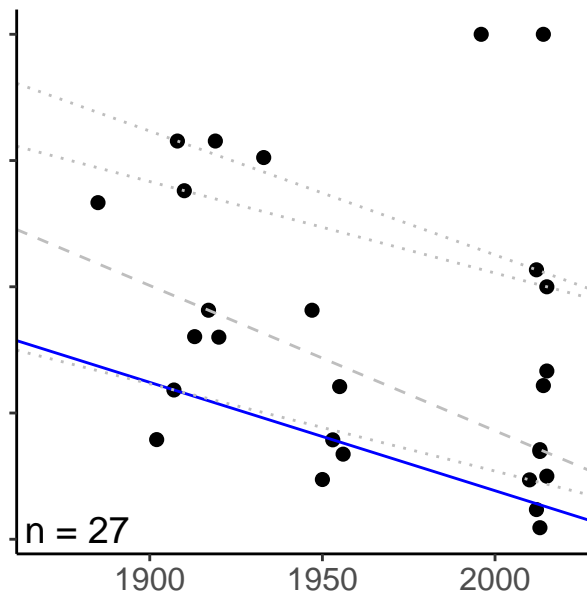

Supplement: Supplemental Information 5 — Weight of fish reported as being exceptionally large in printed news headlines from 1869 to 2015, relative to the maximum species-specific weight, for three species groups: (A) pelagic gamefish (n = 73), (B) oceanic sharks (n = 46), and (C) charismatic megafishes (n = 27). The species included in each group are given in Table S2. Lines represent quantiles regressions that met a minimum sample size (see Methods). Significant quantile regressions are shown as solid lines; non-significant (p < 0.05) quantiles regressions are shown in dotted lines. The dashed line is the 50th quantile. Associated statistics are given in Table S5. [file peerj-07-6395-s005.pdf]

(A) Unknown

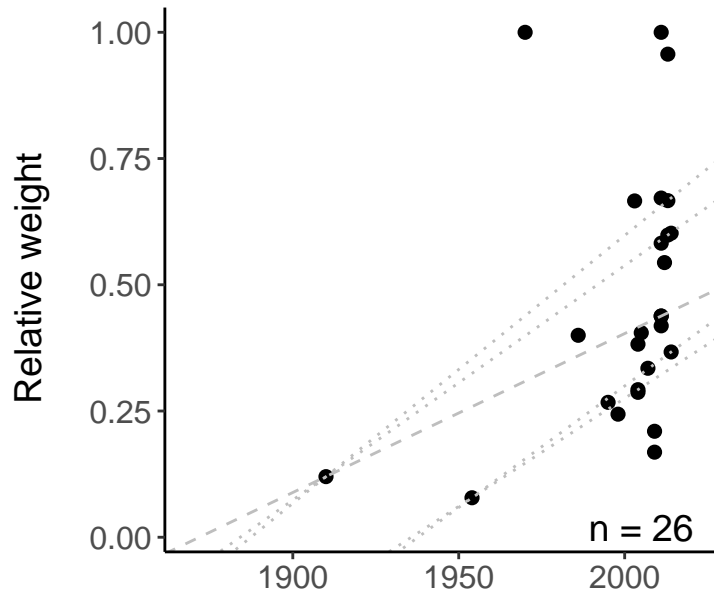

(B) Low risk

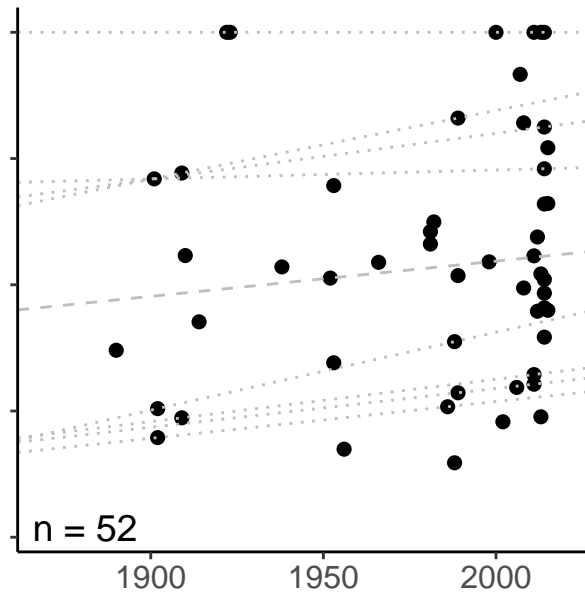

(C) High risk

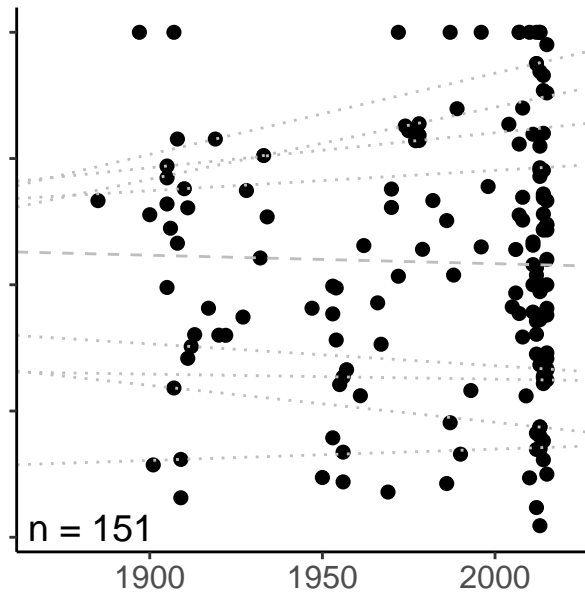

Year

Supplement: Supplemental Information 6 — Weight of fish reported as being exceptionally large in printed news headlines from 1869 to 2015, relative to the maximum species-specific weight, for three categories of extinction risk: (A) unknown risk (i.e., data deficient, not evaluated, least concern; n = 26), (B) low risk (i.e., near threatened and vulnerable; n = 52), and (C) high risk (i.e., endangered and critically endangered; n = 151). Risk of extinction was derived from the current IUCN Red List. The species included in each group are given in Table S2. Lines represent quantiles regressions that met a minimum sample size (see Methods). Significant quantile regressions are shown as solid lines; non-significant (p < 0.05) quantiles regressions are shown in dotted lines. The dashed line is the 50th quantile. Associated statistics are given in Table S6. [file peerj-07-6395-s006.pdf]
