# Supplementary material for: Shifting headlines? Size trends of newsworthy fishes
Source: PeerJ. 2019 Feb 15;7:e6395. doi: 10.7717/peerj.6395 (PMC6378912; doi:10.7717/peerj.6395)
Supplement: Supplemental Information 7 — Methods for creating probability distributions of the relative length (Lhuge/Linf) caught in simulated age-structured populations with varying fishing mortalities (F = 0.05, F = 0.2, F = 0.8) for different sampling efforts (N = 10000 top row, N = 1000 middle row, N = 100 bottom row). Dotted black vertical lines in Figure S3 indicate the mean relative length of 72.1%, observed in news articles compiled in this study; red and blue vertical lines indicate simulated mean relative lengths that are above or below this observed threshold, respectively. [file peerj-07-6395-s007.docx]

**Supplement 5 – Age-structured fisheries model simulation**

**Shifting headlines? Trends in sizes of newsworthy fishes**

Fiona T. Francis, Brett R. Howard, Trevor A. Branch, Adrienne E. Berchtold, Laís C.T. Chaves, Jillian C. Dunic, Brett Favaro, Kyla M. Jeffrey, Luis Malpica-Cruz, Natalie Maslowski, Jessica A. Schultz, Nicola S. Smith, and Isabelle M. Côté

**Age-structured fisheries model simulation**

**Methods**

To create a theoretical distribution of large fish to compare to our observed average relative sizes of newsworthy fishes, we modelled a generic age-structured fish population using predetermined von Bertalanffy growth parameters (Table S8) and a Beverton-Holt stock–recruitment curve. Because the size-frequency distributions of real fish populations are influenced by both fishing pressure and sampling effort (i.e., the number of observations of fish), the simulated population was subjected to three levels of fishing pressure (F = 0.05, 0.2, and 0.8) at three different sampling efforts (10000 individuals, 1000 individuals and 100 individuals). Model simulations identified the largest individual (L_huge_) sampled from the population after 100 years. This was repeated 1000 times, resulting in a distribution of 1000 L_huge_ values for each combination of fishing pressure and sampling effort. These values were used to create a probability distribution of average relative fish lengths (L_huge_/L_inf_). The average relative length calculated for our dataset was then compared against this distribution. We restricted this analysis to length data as these were deemed to be the more reliable measure of size.

**Results**

Average relative lengths from simulated age-structured populations varied depending on fishing pressure and sampling effort. For well-observed populations at all fishing pressures, mean simulated relative length was larger than that reported by the media, i.e. 72% (Fig. S4). In such instances, the observed average relative length of 72% represents fishes that are not particularly large. In contrast, at low sampling effort and higher fishing pressures, the simulated relative lengths were smaller than reported relative length (Fig. S4). Thus, if the fishes in our dataset are from such populations, they are truly newsworthy.


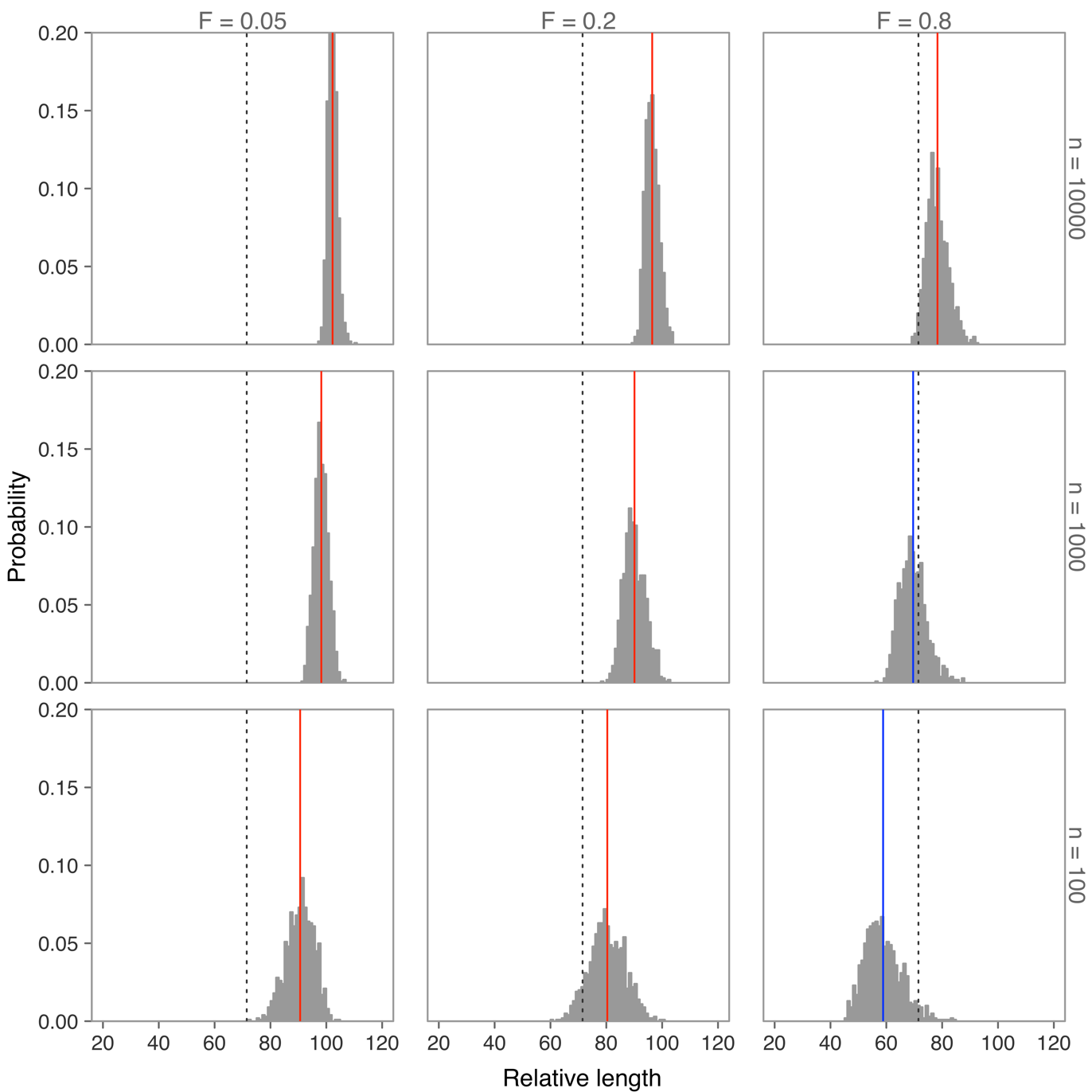


**Figure S4. Probability distributions of the relative length (L_huge_/L_inf_) caught in simulated age-structured populations with varying fishing mortalities (F = 0.05, F = 0.2, F = 0.8) for different sampling efforts** (N = 10000 top row, N = 1000 middle row, N = 100 bottom row). Dotted black vertical lines indicate the mean relative length of 72.1%, observed in news articles compiled in this study; red and blue vertical lines indicate simulated mean relative lengths that are above or below this observed threshold, respectively.

Table S8. List of parameter values used to generate our generic von Bertalanffy population growth curve and Beverton-Holt stock-recruitment curve.

| **Parameter** | **Value** | **Parameter description** |
| --- | --- | --- |
| ***Von Bertalanffy*** | | |
| vbLinf | 100 | asymptotic mean length of very old fish |
| vbK | 0.1 | rate of growth in length of fish |
| Vbt0 | 0 | length of a fish of age 0 |
| CVLinf * | 0.0115 | variability around asymptotic mean length of very old fish |
| CVintercept * | 0.2011 | variability around von Bertalanffy growth curve intercept |
| CVslope * | -0.1749 | variability around rate of growth in length of fish |
| ***Beverton-holt*** | | |
| h | 0.75 | recruitment when spawning biomass is at 20% of unfished stock biomass |
| R0 | 10000 | number of recruits every year in an unfished population |
| M | 0.2 | instantaneous natural mortality |
| Asel | 3 | age at selectivity (knife-edge) |
| Amat | 3 | age at maturity (knife-edge) |

* values based on data from Erzini & Castro 1994 Fishery Bulletin 92:865-871.
